# The Nature of the Dietary Protein Impacts the Tissue-to-Diet ^15^N Discrimination Factors in Laboratory Rats

**DOI:** 10.1371/journal.pone.0028046

**Published:** 2011-11-22

**Authors:** Nathalie Poupin, Cécile Bos, François Mariotti, Jean-François Huneau, Daniel Tomé, Hélène Fouillet

**Affiliations:** 1 INRA, CRNH-IdF, UMR914 Nutrition Physiology and Ingestive Behavior, Paris, France; 2 AgroParisTech, CRNH-IdF, UMR914 Nutrition Physiology and Ingestive Behavior, Paris, France; Institut Pluridisciplinaire Hubert Curien, France

## Abstract

Due to the existence of isotope effects on some metabolic pathways of amino acid and protein metabolism, animal tissues are ^15^N-enriched relative to their dietary nitrogen sources and this ^15^N enrichment varies among different tissues and metabolic pools. The magnitude of the tissue-to-diet discrimination (Δ^15^N) has also been shown to depend on dietary factors. Since dietary protein sources affect amino acid and protein metabolism, we hypothesized that they would impact this discrimination factor, with selective effects at the tissue level. To test this hypothesis, we investigated in rats the influence of a milk or soy protein-based diet on Δ^15^N in various nitrogen fractions (urea, protein and non-protein fractions) of blood and tissues, focusing on visceral tissues. Regardless of the diet, the different protein fractions of blood and tissues were generally ^15^N-enriched relative to their non-protein fraction and to the diet (Δ^15^N>0), with large variations in the Δ^15^N between tissue proteins. Δ^15^N values were markedly lower in tissue proteins of rats fed milk proteins compared to those fed soy proteins, in all sampled tissues except in the intestine, and the amplitude of Δ^15^N differences between diets differed between tissues. Both between-tissue and between-diet Δ^15^N differences are probably related to modulations of the relative orientation of dietary and endogenous amino acids in the different metabolic pathways. More specifically, the smaller Δ^15^N values observed in tissue proteins with milk than soy dietary protein may be due to a slightly more direct channeling of dietary amino acids for tissue protein renewal and to a lower recycling of amino acids through fractionating pathways. In conclusion, the present data indicate that natural Δ^15^N of tissue are sensitive markers of the specific subtle regional modifications of the protein and amino acid metabolism induced by the protein dietary source.

## Introduction

In ecological studies, naturally-occurring stable isotope signatures of animals have been extensively used to track diets and reconstruct trophic relationships within food webs [1]–[3]. Animals are generally ^15^N-enriched over their diet, with an average discrimination factor (Δ^15^N, the difference in ^15^N natural abundance between body and diet) of 3.4‰ [4], which varies among tissues and metabolic pools [4]–[6]. This discrimination originates from the existence of kinetic isotope effects in metabolic pathways involving the bond-breaking or synthesis of nitrogenous compounds, during which the molecules that contain the lighter nitrogen isotope (^14^N) are preferentially utilized to the detriment of the same molecules containing the heavier isotope (^15^N). When located at a metabolic branch point, such kinetic isotope effects induce a preferential flow of ^14^N into the products generated by the reaction with the larger isotope effect, which results in an isotopic fractionation between the metabolic pools produced by the competing reactions, as reflected by a difference in Δ^15^N of these pools [7]. More specifically, deamination and transamination enzymes are likely to preferentially convert amino groups containing ^14^N [8], [9], which is thought to result in the urinary excretion of ^15^N-depleted end products and the incorporation of residual ^15^N-enriched amino acids into the body [10], [11] in line with the body-to-diet positive Δ^15^N. Interestingly, there is a growing body of evidence that the magnitude of Δ^15^N vary between individuals depending on their specific nutritional, physiological or pathological conditions. For instance, the hair ^15^N natural abundance values are good markers of animal protein consumption [12] and vary with pregnancy [13], nutritional stress [14], anorexia [15] and cirrhosis [16] in humans. More specifically, Δ^15^N values in some animal tissues have been shown to vary with protein intake, balance or starvation [6], [14], [15], [17]–[19] as well as with dietary protein source and quality [6], [20], [21]. However, because the latter studies were meta-analyses of literature data over different species from studies where the quantitative protein intake was not controlled for, there is a large risk of confusion between the specific effects of the qualitative and quantitative aspects of protein intake. Indeed, a difference in the dietary protein source is often associated with a difference in the level of protein intake and these two factors may affect Δ^15^N values [22]. Accordingly, dedicated experimental investigations under controlled nutritional conditions are required to examine the changes in the pattern of isotopic discrimination between the animal tissues and the diet that specifically relate to the nature and the quality of the dietary protein source.

The magnitude of Δ^15^N has been proposed to reflect the efficiency of dietary protein assimilation and the importance of amino acid recycling [17], [23], [24], which both vary with the dietary protein source and quality [25]–[27]. In the present work, we hypothesized that dietary protein of different composition and nutritional characteristics would result in different discrimination factors in nitrogen pools. Since different dietary proteins may differentially impact amino acid metabolism in tissues [25], [28], we also hypothesized that the magnitude of the impact of the dietary protein source on nitrogen discrimination factors might vary between tissues. Most studies reporting variations in natural ^15^N abundances depending on nutritional or physiological conditions have focused on pools that can be easily sampled, such as blood or hair [12]–[16], [18], [20], [29]. Therefore, the data are fragmented, and the potential isotopic variations in other active metabolic pools have seldom been described, while such variations may interestingly indicate underlying changes in amino acid and protein metabolism. In this context, multiple simultaneous Δ^15^N measurements taken from different tissues may trace complex changes in inter-organ protein metabolism. In this study, we determined the isotopic nitrogen composition of a large set of pools in rats after their adaptation to two dietary protein sources (milk and soy proteins) differing slightly in their amino acid composition and their effect on the tissue protein metabolism [25], [28].

## Results

### Body composition, tissue mass and nitrogen content

The two experimental groups receiving a diet with either milk protein (MP) or soy protein (SP) had similar growth rates and tissue masses (Table 1). Nitrogen contents of the protein (PF) and non-protein (nPF) fractions of tissues at the end of the 3-wk experimentation period were similar between groups (Table S1), except for a lower nitrogen content in the nPF of the total muscle mass (−37%) in the rats fed with SP compared to MP. None of the total nitrogen content of tissues was impacted by the diet (Table S1). No differences were found in nitrogen content of plasma proteins and urea between groups (Table S1).

**Table 1:** Body weight, tissue masses and plasma variables in rats fed a milk protein- or a soy protein-based diet for 3 wk.

	Milk Protein diet	Soy Protein diet
	(n = 9)	(n = 9)
Initial body weight *(g)*	234±7	234±6
Final body weight *(g)*	361±17	356±17
Tissue mass *(g/100 g BW)*		
Stomach	0.40±0.03	0.37±0.02
Small intestine mucosa	0.71±0.14	0.75±0.20
Liver	2.98±0.12	2.99±0.26
Kidneys	0.65±0.02	0.63±0.04
Gastrocnemius muscle	0.35±0.02	0.36±0.02
Soleus Muscle	0.08±0.01	0.08±0.01
Plasma proteins *(g/L)*	52.4±3.5	55.3±3.8
Plasma urea *(mmol/L)*	6.6±0.4	6.2±1.2

Values are means ± SD. BW, Body Weight. There was no effect of the dietary protein source on tissue masses (Post hoc tests with Bonferroni adjustments, *P*<0.05).

### Variations in Δ^15^N among tissues and proteins

**Figure 1:**
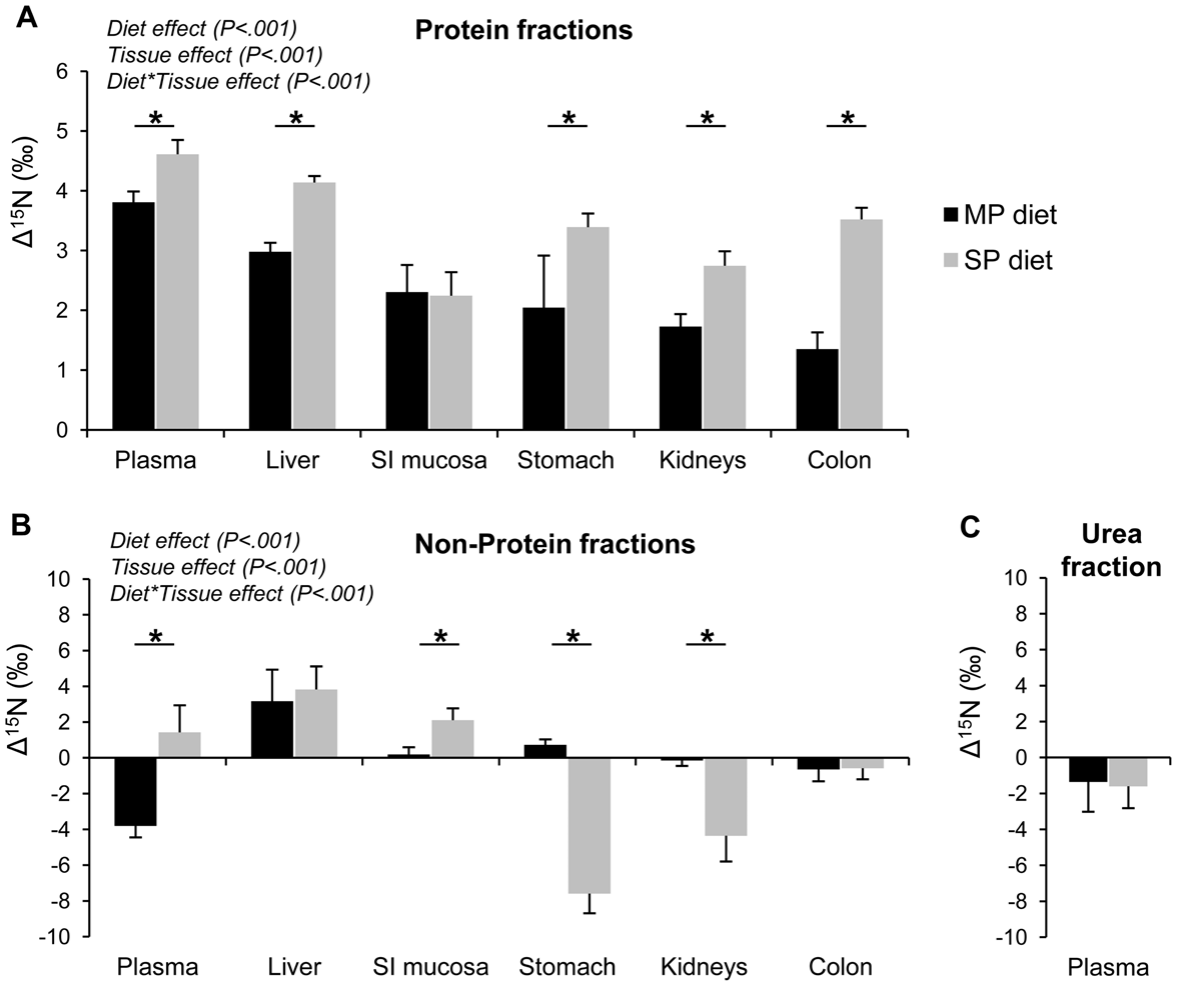
Nitrogen isotopic discrimination values (Δ^15^N) among the sampled tissues of rats. Δ^15^N of (A) tissues protein fractions (δ^15^N_protein fraction_−δ^15^N_diet_), (B) tissue non-protein fractions (δ^15^N_non-protein fraction_−δ^15^N_diet_) and (C) plasma urea (δ^15^N_plasma urea_−δ^15^N_diet_), of rats fed the MP (black bars) and the SP (grey bars) diets. Values are means ± SD. *Effect of the dietary protein source in a given tissue (Post hoc tests with Tukey adjustments, *P*<0.001). All values are significantly different from 0 (Student t-tests, *P*<0.05) except for the non-protein fractions of SI mucosa and kidneys for the MP group. MP, Milk Protein; SP, Soy Protein; SI, Small Intestine.

Using the ^15^N natural abundances (δ^15^N) measured in each sampled pool (Table S2), nitrogen stable isotope discrimination values (Δ^15^N, the difference in δ^15^N between the pool under consideration and the diet) were estimated for the fast turning-over pools that had likely reached, or almost reached, their isotopic equilibrium with the diet at the end of the 3-wk dietary period, and Δ^15^N values were calculated at different levels (whole visceral area, each individual tissue and each nitrogen fraction in a given tissue and in plasma). . At the tissue level, Δ^15^N values were positive and differed greatly among tissues, with liver having the highest ^15^N abundance among the sampled visceral tissues for both groups (Table 2). The Δ^15^N values of tissues were generally close to the Δ^15^N values of their PF, because of the relative amounts of nitrogen in tissue fractions. The Δ^15^N of PF exhibited significant inter-tissues differences (Fig. 1*A*) and ranged from +1.35‰ (±0.25‰) in colon to +3.81‰ (±0.18‰) in plasma in the MP group, and from +2.24‰ (±0.39‰) in small intestine (SI) mucosa to +4.61‰ (±0.24‰) in plasma in the SP group. For both groups, the plasma proteins (liver exported proteins, measured in the vena cava) had the highest Δ^15^N values, and the liver constitutive proteins had Δ^15^N values lower than those of plasma proteins but higher than those of the other visceral proteins (all *Ps*<0.05, Tables S3 & S4).

**Table 2:** Variations in nitrogen isotopic discrimination values among the different tissues and between nitrogen fractions within tissues for rats fed a milk protein- or a soy protein-based diet for 3 wk.

	Milk Protein diet		Soy Protein diet	
	(n = 9)		(n = 9)	
	Δ^15^N	Δ^15^N_PF_- Δ^15^N_nPF_	Δ^15^N	Δ^15^N_PF_- Δ^15^N_nPF_
	‰			
Liver	3.00±0.16^a^	−0.18±1.80	4.13±0.10^a^ *	0.31±1.34
Small intestine mucosa	2.03±0.39^b^	2.12±0.50	2.23±0.40^c^	0.13±0.55*
Stomach	1.86±0.75^b^	1.32±1.00	2.41±0.35^c^	10.99±1.05*
Kidneys	1.59±0.22^bc^	1.88±0.33	2.04±0.34 ^c^ *	7.10±1.44*
Colon	1.23±0.27^c^	1.99±0.50	3.29±0.19^b^ *	4.10±0.67*
Overall (visceral area)	2.58±0.11	1.05±0.98	3.53±0.14*	3.20±1.13*

Weighted isotopic discrimination values (Δ^15^N) were calculated for each visceral tissue (as the weighted average of its protein and non-protein fractions Δ^15^N reported in Fig. 1) and globally for the visceral tissues (as the weighted average of liver, small intestine mucosa, stomach, kidneys and colon Δ^15^N). Differences in Δ^15^N values between protein and non-protein fractions (Δ^15^N_PF_- Δ^15^N_nPF_) were calculated for each tissue. Values are means ± SD.

*Effect of the dietary protein source on tissue Δ^15^N and Δ^15^N_PF_- Δ^15^N_nPF_ values (Post hoc tests with Tukey adjustments, *P*<0.05). All values are significantly different from 0 (Student t-test, *P*<0.05) except for the Δ^15^N_PF_- Δ^15^N_nPF_ values of the small intestine mucosa with the SP diet and of the liver with both diets. Values with different letters in a given column are significantly different (Post hoc tests with Tukey adjustments, *P*<0.05).

### Effect of diet on Δ^15^N in tissues and proteins

Δ^15^N also varied relative to the protein source. At the tissue level, liver, kidney and colon were more enriched in ^15^N in rats fed the SP diet than in rats fed the MP diet, resulting in a higher overall visceral Δ^15^N (3.53‰±0.14‰and 2.58‰±0.11‰ with SP and MP respectively, Table 2). At the protein level, Δ^15^N values were also higher with SP than MP diet in the PF of all tissues except SI mucosa (Fig. 1*A*). These differences between the two diets also varied between tissues. In colon proteins, the Δ^15^N values were ∼160% higher in the SP group than in the MP group (3.52‰ and 1.35‰, respectively), ∼60% higher in kidney proteins (2.75‰ and 1.73‰, respectively) and stomach proteins (3.39‰ and 2.05‰, respectively), ∼40% higher in liver constitutive proteins (4.14‰ and 2.98‰, respectively) and ∼20% higher in liver-exported plasma proteins (4.61‰ and 3.81‰, respectively).

### Variations in Δ^15^N between nitrogen fractions within plasma and tissues

Furthermore, Δ^15^N values differed between nitrogen fractions within plasma (PF vs. urea), and also within tissues (PF vs. nPF). Whatever the diet, plasma urea was depleted in ^15^N relative to the diet and relative to the plasma PF, with no diet effect on the Δ^15^N values of plasma urea (−1.36±1.66‰ and −1.61±1.22‰ in MP and SP groups, respectively). Plasma urea was also ^15^N-depleted relative to the liver nPF, its precursor pool (Fig. 1*B,C*). In tissues, the PF were always enriched in ^15^N relative to the diets (Fig. 1*A*) whereas the nPF had either higher, similar or lower ^15^N abundances compared to the diet depending on both tissue and diet (Fig. 1*B*). In tissues, the PF was generally enriched in ^15^N relative to the nPF (Table 2), except in some tissues were there was no difference between fractions (namely, liver for both MP and SP groups and SI mucosa for the SP group only). Differences in ^15^N abundance between PF and nPF varied from tissue to tissue and between diets (Table 2): they were higher for the SP diet than for the MP diet in colon, kidneys and stomach, whereas they were lower in plasma and SI mucosa.

## Discussion

In this study, we investigated the specific impact of the dietary protein quality (milk vs. soy protein) on the nitrogen isotopic signatures in a large set of tissues and pools of rats under controlled conditions, i.e. under similar conditions of quantitative protein intake, growth and body composition between groups. The discrimination values (Δ^15^N) were calculated in the fast turning-over pools (visceral tissues and plasma) being at, or close to, their isotopic equilibrium with the diet at the end of the 3-wk experimental period. We showed that Δ^15^N vary greatly among pools and are clearly impacted by the nature of the dietary protein in most of the pools. The observed Δ^15^N differences between diets likely result from subtle modulations of the protein and amino acid metabolism induced by the differences in amino acid composition and quality between milk and soy proteins.

### Variations in the discrimination values among nitrogen fractions within tissues and plasma

Contrary to ecological studies, which have generally reported Δ^15^N values for plasma or tissues analyzed as a whole, we here specifically measured the natural ^15^N abundance in different nitrogen fractions of tissues (PF and nPF) and plasma (PF, nPF and urea).

Firstly, our results of Δ^15^N values for the tissue protein fractions, which represent the main part of tissue nitrogen, confirm that tissues, and more specifically tissue proteins, are generally ^15^N-enriched relative to the diet, with a range of Δ^15^N values (from 1.4‰ to 4.6‰) in line with literature values reported for whole tissues in rodents [1], [4], [8], [30]–[36]. Also, we showed that, for a given tissue, Δ^15^N values varied between its nitrogen fractions. PF were generally significantly ^15^N-enriched compared to nPF, which may be explained by an isotope effect along one or several of the metabolic pathways within tissues, such as metabolic interconversions of amino acids, protein synthesis or protein breakdown.

Δ^15^N values also varied between the different nitrogen fractions of plasma, with plasma urea being ^15^N-depleted compared to plasma proteins. Plasma urea was also ^15^N-depleted compared to the liver nPF, i.e. its main precursor pool, confirming the preferential removal of ^15^N-depleted liver amino acids for urea production because of the isotope effect associated to deamination [8]. Nitrogen metabolism in the liver is a branched pathway, with precursor amino acids (liver nPF) being concurrently used for catabolic (urea production) or anabolic (synthesis of liver and plasma proteins) purposes. The isotopic fractionation observed between the end-products of each branch (i.e., ^15^N-depletion of plasma urea relative to hepatic-synthesized proteins) suggests that the amino acids that are not directed toward deamination but used for protein synthesis become ^15^N-enriched, and thereby contribute to the ^15^N-enrichment of the hepatic-synthesized proteins. The magnitude of this fractionation is expected to depend both on the difference between the isotope effects associated to each of the hepatic metabolic pathways and on their relative activity [37], [38]. Although it is a common hypothesis in the literature that deamination is involved in the ^15^N enrichment of body tissues [39], this had not been demonstrated yet and our study is the first to actually report Δ^15^N values for the metabolic pools of the hepatic branched pathway.

### Variations in the discrimination values among body proteins

We observed significant differences in Δ^15^N values among body proteins. For instance, whatever the dietary protein source, the plasma proteins (which mostly originate from synthesis in the liver) had the highest ^15^N abundance compared to all other sampled proteins including the liver constitutive proteins, and the liver proteins had a higher ^15^N abundance than the other sampled proteins. Consistent observations of higher Δ^15^N values in plasma than liver [5], [40] and in liver than kidney [4], [35] were already reported in the literature.

There is probably no simple mechanism that can solely explain the observed differences between tissues Δ^15^N. The isotopic signatures observed in the nitrogen pools of tissues are actually the result of the relative inward and outward fluxes of nitrogen isotopes in the many different pathways involved in the metabolism of proteins and amino acids (protein synthesis, protein degradation, non-protein metabolism, deamination, transamination, transport…), some of which being known to be associated with an isotope fractionating effect [9], [41], [42]. Therefore, variations in Δ^15^N among body proteins may arise from differences in the number and intensity of these metabolic processes (for instance, greater recycling of amino acids from and for tissue protein turnover) and are probably associated with difference in tissues metabolic rates, as previously suggested to explain the greater ^15^N abundance in the liver [31], [32]. Notably, the observed differences in Δ^15^N between tissues cannot be solely ascribed to differences in tissues turnover rates, since the reported Δ^15^N values of the different body proteins did not rank the same way as their known turnover rate and this ranking greatly varied with the protein source. In addition, the observed differences in isotopic composition between the tissue proteins may be linked to their relative composition in amino acids, which exhibit different isotopic abundances [8]. Further investigations are needed to test and delimit the role of these possible mechanisms in producing Δ^15^N differences between the tissue proteins.

### Variations in the discrimination values in body proteins according to the dietary protein source

The dietary protein source strongly affected the nitrogen isotopic discrimination between body proteins and diet, with higher Δ^15^N in rats fed soy than milk proteins for all reported proteins except that of the SI mucosa. Higher Δ^15^N values have already been reported in liver of rats fed with plant vs. animal protein-based diets [1], [8]. More specifically, higher Δ^15^N values were observed in liver [8] and in plasma and jejunum proteins [38] of rats fed with soy vs. milk protein-based diets. We here show that this effect is a general feature, inasmuch as it is consistent over all the sampled visceral protein pools, except the small intestine. The amplitude of Δ^15^N differences between diets varied among tissues, suggesting that the protein metabolism of tissues may be differentially impacted by the dietary protein source.

The differences in amino acid composition between these two dietary proteins, which induce subtle modulations in their metabolic fate and in the endogenous protein metabolism, certainly explain the higher Δ^15^N values in rats fed soy than milk proteins. Studies in humans [26]–[28] and animals [25], [43] have shown that the soy and milk proteins exhibit different overall and regional rates of utilization, the soy protein being less used for postprandial protein anabolism (i.e., lower net protein utilization) than the milk protein. Indeed, in the case of the soy protein, which contains a lower amount of indispensable amino acids, dietary amino acids are more deaminated and a larger proportion of amino acids incorporated in tissue proteins probably originate from endogenous amino acids that have been transaminated or recycled from protein degradation. Because these metabolic pathways are considered as a source of isotopic fractionation [8], [42], the higher nitrogen isotopic discrimination with soy protein confirms the existence of these additional steps in the utilization of soy protein. It can therefore be hypothesized that the best a dietary protein source matches the metabolic demand for indispensable amino acids, the larger is the proportion of dietary amino acids that are utilized for body protein synthesis and, as a result, the closer is the isotopic composition of the tissues to that of the diet. The fact that, in our study, we generally observed higher Δ^15^N values in body proteins of SP-fed rats than MP-fed rats therefore strongly suggests that nitrogen isotopic discrimination increases when the dietary protein efficiency of deposition decreases. This interestingly agrees with the hypothesis of a negative relationship between Δ^15^N and the net protein utilization of the diet protein source that was suggested in the literature from a set of fragmented data in ecological studies [6], [20], [21], [23], where however, several important factors of variation (amount of nitrogen intake, growth rate, physiological condition, etc.) were not controlled for. By contrast, the present study assessed the impact of the dietary protein source on isotopic composition without any potential bias from variations in the protein intake, body composition or tissue mass (Table 1 and S1). Indeed, despite their differences in amino acids composition, the milk and soy proteins were provided in sufficient amounts in the diet (20% of energy) to satisfy the requirements for indispensable amino acids, as shown by the similar growth and tissue protein accretion in the two dietary groups. The existence of a relation between Δ^15^N and dietary protein deposition efficiency is of significant importance because it indicates that the natural nitrogen isotopic enrichment pattern of tissues could be used as an indicator of the local anabolic use of dietary protein, and thus complements the data regarding total protein metabolism as obtained by classical tracer studies [44], [45].

A limitation of this study is that the experimental diet was not provided over a long enough period of time to allow the slow-turning over, peripheral tissues to reach their isotopic equilibrium with the diet, so that we were only able to investigate the effect of the dietary protein source on tissue-to-diet discrimination values in the fast-turning over tissues, i.e. visceral organs and plasma. Indeed, the rate at which a tissue incorporates the isotopic signature of a diet varies among tissues and is slower for tissues with slow protein turnover rates and/or low growth rates [22]. For instance, more than 6 months are required to ensure nitrogen isotope equilibrium in muscle or fur in adult, non-growing rats [33]. We therefore did not calculate the Δ^15^N in peripheral tissues as, after 3 wk, these values would still be impacted by the initial isotopic state, so that differences between diets in such Δ^15^N values could not be solely attributed to a diet effect on protein metabolism and would thus be difficult to interpret. By contrast, in fast-turning over pools like liver or plasma, Δ^15^N reaches a plateau after 2 weeks [8], [46] or is near equilibrium after 3 weeks [33], as shown by data from diet-shift experiments in rodents. Concerning the other fast-turning over tissues like kidney, we found very few data of isotopic incorporation rates in the literature and the reported values often vary among studies according to the experimental conditions and growth rates [33], [47]. In the present study, we believe that all the visceral tissues were very close to their isotopic equilibrium with the diet after 3-wk, given their high protein turnover rates and the high growth rate of the rats, which should have shortened the equilibration time [19]. Longer-term studies would be of interest to investigate, on a wider range of tissues, the effect of the dietary protein source that we here evidenced on visceral tissues.

In conclusion, the ^15^N discrimination values varied among tissues, between the nitrogen fractions (PF, nPF and urea) and according to the dietary protein source. We showed consistently higher Δ^15^N values in virtually all visceral proteins of rats fed soy than in those fed milk proteins and interpreted this higher discrimination with soy than milk proteins as resulting from a lower dietary protein anabolic use and higher endogenous protein mobilization and recycling of amino acids. In view of these results, nitrogen stable isotope signatures of body proteins appear as highly sensitive markers of the delicate nutritional modulations of protein and amino acid metabolism. As far as we know, our work is the first demonstration that subtle tissue-specific modulations of protein metabolism induced by different dietary proteins and without any detectable effect on tissue composition actually leave a marked selective isotopic footprint in tissues. This novel approach, based on the investigation of the isotopic signatures of metabolic pools at natural abundance level, opens new, exciting perspectives to better characterize and understand the amino acid and protein metabolism. Some interesting mass-balance models have been developed to understand natural variations in Δ^15^N at the level of the whole organism or its lean mass [17], [19], [48], and further modeling studies are still needed to mechanistically understand inter-tissue and inter-fraction variations in nitrogen isotopic composition.

## Materials and Methods

### Ethics Statement

Experiments were carried out in accordance with the recommendations in the NIH Guide for the Care and Use of Laboratory Animals. The protocol was approved by the Ethics Committee for Animal Experiments (COMETHEA) of the Jouy-en-Josas INRA centre and AgroParisTech (Approval Number 11/015).

### Animals and diets

Male Wistar Rats (n = 18), initially weighing 191 g, were purchased from Harlan (France) and housed in a temperature-regulated room (22±2°C) on a 12-h light-dark cycle (dark period from 9:00 to 21:00). Before the beginning of the experiment, the rats were adapted to the laboratory conditions for one wk with free access to a commercial laboratory diet (δ^15^N≈4‰). Animals were then randomly divided into two groups and assigned to receive one of the two experimental diets. Initial body weights were similar for the two groups.

The two diets were isoenergetic, identical in their nutrient composition (20% of energy as protein, 30% as lipids and 50% as carbohydrates) and met the requirements for growth. They differed in their protein source, either milk protein (MP, n = 9) or soy protein (SP, n = 9) (Table 3). Diets were prepared by the UPAE (Unité de Préparation des Régimes Expérimentaux, French National Institut of Agronomic Research, INRA, Jouy en Josas, France).

**Table 3:** Composition of diets.

Component	Milk Protein Diet	Soy Protein Diet
	(n = 9)	(n = 9)
	*g/kg DM*	
Total milk protein	199.3	
Soy protein		199.3
Cornstarch	428.2	433.7
Sucrose	33.6	45.8
Lactose	17.4	
Soybean oil	127.7	132.9
Dairy fat	5.0	
Cellulose	52.8	53.6
AIN-93M-MX mineral mixture	36.9	37.5
AIN-93-VX vitamin mixture	10.6	10.7
Choline	2.4	2.5

DM, Dry Matter.

### Experimental protocol and sampling procedures

Following the 1-wk adaptation period, the rats were fed one of the two experimental diets for 3 wk. Rats were given free access to food from 9:00 to 19:00 (during the dark period) and had free access to water throughout the day. Body weight was measured twice weekly. At the end of week #3 (day 21 or 22), all the rats were killed after an overnight fast.

The rats were anesthetized with an intraperitoneal injection of sodium pentobarbital (100 mg/kg of BW, a dose which is too small to significantly affect the measured isotopic signatures based on the mode of administration of this anesthetic and on distribution kinetics of barbiturates in tissues and plasma of rats [49]). The abdomen was opened and approximately 5 ml of blood were quickly withdrawn from the vena cava. The animals were then killed by rupture of the vena cava and aorta. The liver, small intestine (SI), stomach, colon, kidneys, gastrocnemius and soleus muscles were rapidly dissected out, rinsed, weighed and snap frozen in liquid nitrogen. The SI was scraped to collect the mucosa, which was frozen separately. Hair and skin were sampled. All samples were stored at −20°C until analysis.

### Samples preparation

In each tissue, the protein fraction (PF) and the non-protein fraction (nPF) were isolated for separate determination of nitrogen content and isotopic ratios in each fraction. Frozen tissues were pulverized with a mortar and pestle cooled in liquid nitrogen and precipitated by adding 700 µL of 5-sulfosalicylic acid (10%) per 100 mg of tissue. After centrifugation (2,500 g, 4°C, 15 min), the supernatant was extracted and the pellet was rinsed twice with 700 µL of 5-sulfosalicylic acid (10%). The soluble fraction (i.e., nPF containing the free amino acids) and the insoluble fraction (i.e., PF containing the protein-bound amino acids) were then freeze-dried.

In plasma, the different nitrogen fractions (PF, nPF and urea) were also separated. PF was isolated by precipitation with sulfosalicylic acid (200 µL, 1 g/mL). After 1-h storage at 4°C and centrifugation (2,000 g, 4°C, 20 min), the pellet was rinsed with 1 mL sulfosalicylic acid (1 g/mL), centrifugated again and freeze-dried. The extracted soluble fraction -containing nPF and urea- was neutralized and transferred onto 0.5 mL cation exchange resin (Dowex AG50X8, Biorad, Marnes-la-coquette, France) to bind the ammonium released from urea hydrolysis (8 µL urease for 2 h at 30°C). The ammonium-loaded resin was washed and kept at 4°C until analysis, while the urea-depleted supernatant, containing amino acids and peptides, was collected and filtered to eliminate peptides larger than 3 kDa (Amicon Ultra-4, Ultracel 3k, Millipore, Carrigtwohill, Ireland) and isolate nPF. Before isotopic determination, urea-derived-ammonia was eluted from the resins by the addition of KHSO_4_ (2.5 mol/L).

### Elemental analysis and isotopic determinations

The natural stable isotope ratios of nitrogen were determined in plasma urea and in PF and nPF of tissues and plasma using an isotope-ratio mass spectrometer (Isoprime, VG Instruments, Manchester, UK) coupled to an elemental analyzer (EA 3000, Eurovector, Italy). Standards were included in every run to correct for the possible variations in raw values measured by the mass spectrometer. Results were expressed using the delta notation according to the following equation:
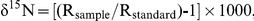
where R_sample_ and R_standard_ are the nitrogen isotope ratio of the heavier isotope to the lighter isotope (^15^N/^14^N) for the sample being analyzed and the internationally defined standard (atmospheric N_2_, R_standard_ = 0.0036765), respectively, and δ is the delta notation in parts per 1000 or per mill (‰) relative to the standard.

The total nitrogen content of the tissues PF and nPF and of the plasma PF were determined using the elemental analyzer (EA 3000, Eurovector, Italy), with atropine as standard. Urea concentrations in plasma were determined with a commercial kit using an enzymatic method (Urea kit S-1000, Biomerieux, Craponne, France).

### Calculations and statistics

Tissue samples were weighed before and after freeze-drying to estimate tissue dry matter. The nitrogen content of each sampled tissue fraction (PF or nPF) was calculated on the basis of the following equation:
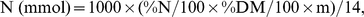
where %N and %DM (%) are respectively the nitrogen and the dry matter percentages measured in the sample, and m (g) is the total mass of the tissue. Total muscle and skin masses were not measured, but were estimated as 45% [50] and 15% [51] of total body mass, respectively.

The nitrogen content of plasma PF and nPF was calculated as the product of the nitrogen concentration in the fraction and the plasma volume, estimated as 3.5% of body mass [52]. The nitrogen concentration in the plasma nPF was estimated using aminoacidemia values obtained by our group in similar conditions (3.1 mmol/L i.e., 4.4 mmol/L of nitrogen equivalents) [53]. The nitrogen content of the plasma urea pool (N_urea_, mmol) was assessed on the basis of the plasma urea concentration (C_urea_, mmol/L of urea nitrogen), its volume of distribution (i.e., total body water, estimated as 62.3% of the body mass, BM, Kg) [54], and a correction factor of 92% which represents the water content of the blood: 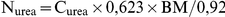 [55].

Isotopic nitrogen discrimination between a pool and the diet was described as the difference in δ^15^N using the Δ notation, where Δ^15^N = δ^15^N_pool_−δ^15^N_diet_ (Δ^15^N>0 indicates a greater abundance in ^15^N in the pool relative to the diet). As MP and SP diets had different natural isotopic ratios (δ^15^N_diet_ were 7.7‰ for MP and 1.7‰ for SP), Δ^15^N is used to compare the isotopic composition of tissues of animals fed different diets.

Δ^15^N values were calculated in the fast turning-over pools having likely reached, or almost reached, their isotopic equilibrium with the diet at the end of the 3-wk experimental period (plasma urea and each of the PF and nPF of visceral tissues and plasma). For each visceral tissue and plasma, an overall Δ^15^N value was calculated as the weighted average of the isotopic compositions of its different nitrogen fractions, as 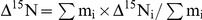, where m_i_ is the amount of nitrogen in the fraction i and Δ_i_ is the isotopic discrimination between the fraction i and the diet. Likewise, a pooled visceral Δ^15^N was calculated as the weighted average of the isotopic compositions of the PF and nPF of liver, SI mucosa, stomach, kidneys and colon.

Results are expressed as means ± SD. The effects of diet (MP versus SP) and tissue and their interaction were analyzed by a two-way analysis of variance (Proc GLM, SAS 9.1, SAS Institute, Cary, NC, USA). Post hoc tests with Tukey or Bonferroni adjustments were used for multiple comparisons between tissues and dietary groups (MP versus SP). *P* values≤0.05 were considered significant.

## Supporting Information

Table S1 Nitrogen composition of tissues and plasma of rats fed a milk protein- or a soy protein-based diet for 3 wk.
(DOC)

Table S2 ^15^N natural abundances (δ^15^N) in the different nitrogen fractions of tissues and plasma.
(DOC)

Table S3 Statistical significances of differences in discrimination values (Δ^15^N) between body proteins in rats fed a milk protein-based diet for 3 wk.
(DOC)

Table S4 Statistical significances of differences in discrimination values (Δ^15^N) between body proteins in rats fed a soy protein-based diet for 3 wk.
(DOC)
