# Supplementary material for: The Nature of the Dietary Protein Impacts the Tissue-to-Diet 15N Discrimination Factors in Laboratory Rats
Source: PLoS One. 2011 Nov 22;6(11):e28046. doi: 10.1371/journal.pone.0028046 (PMC3222673; doi:10.1371/journal.pone.0028046)
Supplement: Table S1 — Nitrogen composition of tissues and plasma of rats fed a milk protein- or a soy protein-based diet for 3 wk. (DOC) [file pone.0028046.s001.doc]

**Supplemental Table 1:** Nitrogen composition of tissues and plasma of rats fed a milk protein- or a soy protein-based diet for 3 wk.

|  | | **Milk Protein Diet**  **(n=9)** | | | | | |  | **Soy Protein Diet**  **(n=9)** | | | | | |
| --- | --- | --- | --- | --- | --- | --- | --- | --- | --- | --- | --- | --- | --- | --- |
|  | | **Protein Fraction** | | **Non-Protein Fraction** | | **Total Nitrogen** | |  | **Protein Fraction** | | **Non-Protein Fraction** | | **Total Nitrogen** | |
|  | *mmol N / 100g BW* | | | | | | | | | | | | |  |
| **Visceral tissues** |  | |  | |  | |  | |  |  | |  | |  |
| Liver | 7.11 ± 0.45 | | 0.32 ± 0.09 | | 7.44 ±0.47 | |  | | 6.72 ±0.26 | 0.33 ± 0.06 | | 7.05 ± 0.24 | |  |
| SI mucosa | 0.87 ± 0.15 | | 0.13 ± 0.04 | | 1.00 ±0.18 | |  | | 0.81 ±0.20 | 0.12 ± 0.05 | | 0.93 ± 0.24 | |  |
| Stomach | 0.60 ± 0.15 | | 0.09 ± 0.03 | | 0.69 ±0.15 | |  | | 0.68 ±0.07 | 0.07 ± 0.01 | | 0.74 ± 0.07 | |  |
| Kidneys | 1.28 ± 0.05 | | 0.11 ± 0.04 | | 1.39 ±0.05 | |  | | 1.27 ±0.10 | 0.14± 0.04 | | 1.41 ± 0.11 | |  |
| Colon | 0.50 ± 0.08 | | 0.03 ± 0.01 | | 0.53 ±0.09 | |  | | 0.46 ±0.06 | 0.03 ± 0.004 | | 0.48 ± 0.06 | |  |
| **Peripheral tissues** |  | |  | |  | |  | |  |  | |  | |  |
| G. muscle | 0.85 ± 0.04 | | 0.07 ± 0.02 | | 0.91 ±0.05 | |  | | 0.86 ±0.06 | 0.07 ± 0.01 | | 0.93 ± 0.07 | |  |
| S. muscle | 0.20 ± 0.02 | | 0.04 ± 0.02 | | 0.24 ±0.04 | |  | | 0.21 ±0.01 | 0.02 ± 0.004 | | 0.23 ± 0.02 | |  |
| Total muscles† | 109.3 ± 2.9 | | 14.0 ± 4.0 | | 123.3 ±5.3 | |  | | 112.0 ±4.2 | 8.9 ± 1.0* | | 120.8 ± 4.0 | |  |
| Skin† | 50.1 ± 4.1 | | 1.2 ± 0.3 | | 51.3 ±4.0 | |  | | 46.3 ±5.9 | 1.1 ± 0.3 | | 47.5 ± 5.8 | |  |
| Red blood cells | 10.4 ± 0.3 | | 0.09 ± 0.023 | | 10.5 ±0.3 | |  | | 10.6 ±1.0 | 0.08 ± 0.02 | | 10.7 ± 1.0 | |  |
| **Plasma** |  | |  | |  | |  | |  |  | |  | |  |
| Urea**†** |  | |  | | 0.90 ±0.06 | |  | |  |  | | 0.83 ± 0.16 | |  |
| Proteins | 2.10 ± 0.14 | |  | |  | |  | | 2.21 ±0.15 |  | |  | |  |

Values are means ± SD. BW, Body Weight; SI, Small Intestine; G., Gastrocnemius; S., Soleus; N., Nitrogen. †estimated values (see text for explanations). *Effect of the dietary protein source for a given fraction type (Post hoc tests with Bonferroni adjustments, *P* < 0.05)
