# Supplementary material for: The Nature of the Dietary Protein Impacts the Tissue-to-Diet 15N Discrimination Factors in Laboratory Rats
Source: PLoS One. 2011 Nov 22;6(11):e28046. doi: 10.1371/journal.pone.0028046 (PMC3222673; doi:10.1371/journal.pone.0028046)
Supplement: Table S2 — 15N natural abundances (δ15N) in the different nitrogen fractions of tissues and plasma. (DOC) [file pone.0028046.s002.doc]

**Supplemental Table 2: ^15^N natural abundances (δ^15^N) in the different nitrogen fractions of tissues and plasma**

|  | **Milk Protein Diet**  **(n=9)** | | |  | **Soy Protein Diet**  **(n=9)** | | |
| --- | --- | --- | --- | --- | --- | --- | --- |
|  | **Protein Fraction** | **Non-Protein Fraction** | **Total Nitrogen** |  | **Protein Fraction** | **Non-Protein Fraction** | **Total Nitrogen** |
|  |  |  |  | *‰* |  |  |  |
| **Visceral Tissues** |  |  |  |  |  |  |  |
| Liver | 10.68 ± 0.15 | 10.86 ± 1.77 | 10.70 ± 0.16 |  | 5.80 ± 0.11 | 5.49 ± 1.29 | 5.79 ± 0.10 |
| SI mucosa | 10.01 ± 0.45 | 7.89 ± 0.40* | 9.73 ± 0.39 |  | 3.90 ± 0.39 | 3.77 ± 0.65 | 3.89 ± 0.40 |
| Stomach | 9.75 ± 0.87 | 8.42 ± 0.31* | 9.56 ± 0.75 |  | 5.05 ± 0.23 | -5.94 ± 1.00* | 4.07 ± 0.35 |
| Kidneys | 9.43 ± 0.21 | 7.55 ± 0.31* | 9.29 ± 0.22 |  | 4.41 ± 0.24 | -2.70 ± 1.44* | 3.70 ± 0.34 |
| Colon | 9.05 ± 0.28 | 7.06 ± 0.67* | 8.93 ± 0.27 |  | 5.18 ± 0.19 | 1.08 ± 0.62* | 4.95 ± 0.19 |
| **Peripheral Tissues** |  |  |  |  |  |  |  |
| G. muscle | 8.79 ± 0.12 | 3.86 ± 0.44* | 8.42 ± 0.13 |  | 4.19 ± 0.11 | 0.40 ± 0.22* | 3.91 ± 0.08 |
| S. muscle | 8.79 ± 0.21 | 3.79 ± 0.39* | 8.01 ± 0.46 |  | 4.39 ± 0.22 | -0.03 ± 0.50* | 4.07 ± 0.24 |
| Skin | 8.42 ± 0.25 | 6.92 ± 1.11* | 8.39 ± 0.24 |  | 5.50 ± 0.17 | 1.31 ± 0.48* | 5.40 ± 0.18 |
| Red blood cells | 7.41 ± 0.31 | 4.97 ± 1.58* | 7.39 ± 0.31 |  | 5.03 ± 0.20 | 5.51 ± 4.10 | 5.03 ± 0.22 |
| Hair |  |  | 7.64 ± 0.34 |  |  |  | 4.93 ± 0.32 |
| **Plasma** |  |  |  |  |  |  |  |
| Urea |  |  | 6.34 ± 1.66 |  |  |  | 0.05 ± 1.22 |
| Other | 11.51 ± 0.18 | 3.89 ± 0.65* | 11.45 ± 0.18 |  | 6.27 ± 0.24 | 3.09 ± 1.51* | 6.25 ± 0.25 |

δ^15^N (‰) in rats fed a milk protein (δ^15^N_diet_ = 7.7 ‰) or a soy protein (δ^15^N_diet_ = 1.7 ‰) based diet for 3 wk. Values are means ± SD. SI, Small Intestine; G., Gastrocnemius; S., Soleus. *Effect of the fraction type for a given diet (Post hoc tests with Bonferroni adjustments, *P* < 0.05).
