# Supplementary material for: The Nature of the Dietary Protein Impacts the Tissue-to-Diet 15N Discrimination Factors in Laboratory Rats
Source: PLoS One. 2011 Nov 22;6(11):e28046. doi: 10.1371/journal.pone.0028046 (PMC3222673; doi:10.1371/journal.pone.0028046)
Supplement: Table S3 — Statistical significances of differences in discrimination values (Δ15N) between body proteins in rats fed a milk protein-based diet for 3 wk. (DOC) [file pone.0028046.s003.doc]

**Supplemental Table 3: Statistical significances of differences in discrimination values (Δ^15^N) between body proteins in rats fed a milk protein-based diet for 3 wk (n=9)**

|  | Liver | SI mucosa | Stomach | Kidneys | Colon |
| --- | --- | --- | --- | --- | --- |
| Plasma | ** | *** | *** | *** | *** |
| Liver |  | * | *** | *** | *** |
| SI Mucosa |  |  | NS | NS | *** |
| Stomach |  |  |  | NS | * |
| Kidneys |  |  |  |  | NS |

*P*-values were calculated using pairwise comparisons between tissues with Tukey’s adjustment for multiple comparisons. **P*-value < 0.05; ***P*-value < 0.01; ****P*-value < 0.001. SI, Small Intestine.
